# CALU promotes lung adenocarcinoma progression by enhancing cell proliferation, migration and invasion

**DOI:** 10.1186/s12931-024-02901-3

**Published:** 2024-07-05

**Authors:** Yan Li, Shengnan Sun, Hui Zhang, Yongjian Jing, Xingzhao Ji, Qiang Wan, Yi Liu

**Affiliations:** 1 Department of Pulmonary and Critical Care Medicine, The Second Hospital of Shandong University, Jinan, Shandong 250033 China; 2 Key Laboratory of Cell Metabolism in Medical and Health of Shandong Provincial Health Commission, Central Hospital Affiliated to Shandong First Medical University, Jinan, Shandong 250021 China; 3 Department of Pulmonary and Critical Care Medicine, Shandong Provincial Hospital, Shandong University, Jinan, Shandong 250021 China; 4 The First People’s Hospital of Pingyuan County, Dezhou, Shandong 253100 China; 5 Department of Pulmonary and Critical Care Medicine, Shandong Provincial Hospital, Shandong First Medical University, Jinan, Shandong 250021 China

**Keywords:** Calumenin, Lung adenocarcinoma, Proliferation, Migration, Ingenuity pathway analysis

## Abstract

**Background:**

Lung cancer is the second most common cancer with the highest mortality in the world. Calumenin as a molecular chaperone that not only binds various proteins within the endoplasmic reticulum but also plays crucial roles in diverse processes associated with tumor development. However, the regulatory mechanism of calumenin in lung adenocarcinoma remains elusive. Here, we studied the impact of calumenin on lung adenocarcinoma and explored possible mechanisms.

**Methods:**

5-ethynyl-2’-deoxyuridine assay, colony formation, transwell and wound healing assays were performed to explore the effects of calumenin on the proliferation and migration of lung adenocarcinoma cells. To gain insights into the underlying mechanisms through which calumenin knockdown inhibits the migration and proliferation of lung adenocarcinoma, we performed Gene Ontology, Kyoto Encyclopedia of Genes and Genomes, Gene Set Enrichment Analysis and Ingenuity Pathway Analysis based on transcriptomics by comparing calumenin knockdown with normal A549 cells.

**Results:**

The mRNA and protein levels of calumenin in lung adenocarcinoma are highly expressed and they are related to an unfavorable prognosis in this disease. Calumenin enhances the proliferation and migration of A549 and H1299 cells. Gene Set Enrichment Analysis revealed that knockdown of calumenin in A549 cells significantly inhibited MYC and V-Ki-ras2 Kirsten rat sarcoma viral oncogene homolog signaling pathways while activating interferon signals, inflammatory signals, and p53 pathways. Ingenuity pathway analysis provided additional insights, indicating that the interferon and inflammatory pathways were prominently activated upon calumenin knockdown in A549 cells.

**Conclusions:**

The anti-cancer mechanism of calumenin knockdown might be related to the inhibition of MYC and KRAS signals but the activation of interferon signals, inflammatory signals and p53 pathways.

**Supplementary Information:**

The online version contains supplementary material available at 10.1186/s12931-024-02901-3.

## Background

Lung cancer is the second most common cancer with the highest mortality in the world [1]. Non-small cell lung cancer (NSCLC), accounting for 85% of lung cancer, and lung adenocarcinoma (LUAD) is the most common NSCLC, accounting for nearly 60% of NSCLC [2] with a five-year survival rate of less than 20% for terminal LUAD [3]. The current therapeutic strategies for LUAD comprise surgery, chemotherapy, radiotherapy, targeted therapy, immunological therapy, or their combination [4, 5]. Despite some strides made in the treatment landscape, a substantial proportion of LUAD cases still lack viable therapeutic options. Consequently, the identification of biomarkers and drug targets is of great scientific significance for the construction of precise treatment strategies for LUAD.

Calumenin (CALU) is a mammalian low-affinity calcium-binding protein [6] that possesses multiple EF-hand motifs and belongs to the CREC (Ca ^2+^ -binding protein of 45 kDa, reticulocalbin, ER Ca ^2+^ -binding protein of 55 kDa, calumenin, CREC) family [7, 8]. In humans, CALU is primarily localized in the endoplasmic reticulum and Golgi apparatus. The cDNA sequence of CALU includes an N-glycosylation site at Asn-131, and studies have demonstrated that CALU undergoes glycosylation and secretion from cultured cells [9]. Given its distribution in the endoplasmic reticulum, Golgi apparatus, and secretory pathway, CALU may exhibit dual functions both intracellularly and extracellularly. Mass spectrometry analysis has revealed phosphorylation sites at Ser44 and Thr62 of CALU [10, 11]. Moreover, Shah [12] have identified CALU as a target for tyrosine phosphorylation through chemical genetic screening of v-src substrates. Numerous investigations have highlighted CALU as a molecular chaperone that not only binds various proteins within the endoplasmic reticulum [13] but also plays crucial roles in calcium cycling, vascular calcification [14], thrombosis [15], cell migration [16], apoptosis [17], and diverse processes associated with tumor development [18].

Through database mining, microarray analysis, proteomics, transcriptomics, and other approaches, several studies [19–23] have unveiled the elevated expression of CALU in lung cancer, colon cancer, glioma, and breast cancer, positioning it as a promising biomarker. For instance, diagnostic accuracy estimation of differentially expressed genes showed that a gene panel comprising CALU can effectively distinguish LUAD tumors from healthy samples. Furthermore, cancer patients with aberrant expression of these signature genes exhibit significantly lower survival rates compared to other patients. However, no further experimental validation has been conducted to date. Considering the unique physiological role of CALU, it is reasonable to postulate that it could exert a substantial impact on LUAD. In our present study, we employed bioinformatics analysis techniques and vitro experiments to investigate the potential role of CALU in LUAD. To gain insights into the underlying mechanisms through which CALU knockdown inhibits the migration and proliferation of LUAD, we performed Gene Ontology (GO), Kyoto Encyclopedia of Genes and Genomes (KEGG), Gene Set Enrichment Analysis (GSEA) and Ingenuity Pathway Analysis (IPA) based on transcriptomic results by comparing CALU knockdown with normal A549 cells. CALU can promote the proliferation, migration and invasion of LUAD cells, which may be a key target and play an extremely important role in inhibiting the progression of LUAD.

## Materials and methods

### Human tissue samples

Human tissue samples, including six lung adenocarcinoma samples, were collected during pneumonectomy or lobectomy procedures at Jinan Central Hospital between 2020 and 2022. These samples were obtained from patients who had not received preoperative chemotherapy or radiotherapy. The study protocol was approved by the Ethics Committee of Jinan Central Hospital Affiliated to Shandong First Medical University, with the Ethics Approval Number SZR2022-015-01. All procedures were conducted in compliance with the applicable guidelines and regulations.

### Cell lines

The cell lines used in this study included normal human bronchial epithelial cells (HBE), purchased from American Type Culture Collection (ATCC) and various lung adenocarcinoma cell lines, namely HCC827, H1299, H1975, A549, PC9, H299 and 95D purchased from Procell (Wuhan, China). Most cell lines were cultured in RPMI-1640 medium (GIBCO, USA), while PC9, A549, and human embryonic kidney 293T cells (HEK 293T cells) were cultured in high glucose DMEM (GIBCO, USA). All culture media were supplemented with 10% fetal bovine serum (GIBCO, USA) and maintained at 37 °C with 5% CO2.

### Transfection

A549 and H1299 cell lines were utilized for transfection experiments. Prior to transfection, these cell lines were passaged in 6-well plates with appropriate growth medium. Lipofectamine 3000 and p3000 (Ribobio, Guangzhou, China) were used for transfection of CALU-siRNA (Ribobio, Guangzhou, China) or overexpressing CALU plasmid (Ribobio, Guangzhou, China). The target sequences of siRNA were siRNA1-5’-GAAGGACCGTGTACATCAT-3’, siRNA2-5’-GACCTTTGATCAGCTGACA-3’, siRNA3-5’-GTGACAAGGTTCACAATGA-3’. Following the addition of Lipofectamine-siRNA or Lipofectamine- p3000-CALU plasmid complex, cells were incubated for 6 h, after which the medium was replaced with fresh experimental medium. Subsequently, the cells were further incubated for an additional 24 h. Post-incubation, cellular harvest was performed using radio immunoprecipitation assay (RIPA) (Solarbio, Beijing, China) buffer supplemented with phenylmethylsulfonyl fluoride (PMSF) (Solarbio, Beijing, China), sodium vanadate, and a protease inhibitor cocktail for subsequent western blotting analysis. Alternatively, TRIzol reagent was used for RNA extraction to facilitate PCR analysis.

### RNA extraction and real-time polymerase chain reaction (qRT-PCR)

Total RNA was extracted from frozen kidneys using the TRIzol reagent (Ambion, USA) following the manufacturer’s protocol. The concentration of RNA was determined using a Nanodrop spectrophotometer. Complementary DNA (cDNA) was synthesized using the PrimeScript RT Reagent Kit (Takara, Beijing, China). Gene expression quantification was performed using the SYBR Green PCR kit (Accurate Biology, Hunan, China) with 10 ng of cDNA. The forward and reverse primers for CALU were 5ʹ-TGCCTTGAGCAAACCCACAG-3ʹ and 5ʹ-CCTTGCTCTCTTCTGGTGTCA-3ʹ, respectively. The primers for ACTIN were 5ʹ-CATGTACGTTGCTATCCAGGC-3ʹ (forward) and 5ʹ-CTCCTTAATGTCACGCACGAT-3ʹ (reverse) (Jinweizhi, Wuhan, China). The qPCR was performed on an ABI 7500 instrument using the 2× Universal PCR Master Mix (Applied Biosystems). The expression levels were analyzed using the ΔΔCt method and normalized to the levels of ACTIN.

### Western blotting

Protein expression in harvested cell lines was examined through SDS-PAGE and western blotting. Samples with equivalent protein concentrations, determined utilizing the bicinchoninic acid (BCA) protein kit (Beyotime, Shanghai, China) were mixed with sample buffer and heated at 98 °C for 5 min. The prepared samples were loaded onto 12% SDS-PAGE gels. PageRuler Marker was purchased from (Thermo Fisher, USA). The separated protein lysates were transferred to PVDF membranes (Millipore, USA) using the semi-dry transfer method. After blocking with TBS-T, the membranes were incubated overnight at 4 °C with primary antibodies of interest diluted in TBS-T supplemented with 5% Bovine Serum Albumin (BSA) (Solarbio, Beijing, China). Rabbit polyclonal anti-calumenin and anti-GAPDH were purchased from Proteintech (PTG, USA). Following three washes, the membranes were incubated with HRP-conjugated secondary antibodies at a dilution of 1:1000 for 1 h at room temperature. Immunoreactive target bands were visualized using an enhanced chemiluminescence (ECL) system (Tanon5200Multi).

### Wound healing assay

A549 and H1299 cells were cultured until reaching confluency. To create a linear wound, a pipette tip was used to scrape across the confluent cell layer. The cells were subsequently washed twice to remove any detached cells and debris. The size of the wounds was observed and measured at designated time points (0 h and 36 h).

### Transwell assay

Migration and invasion assays were performed. For the A549 or H1299 cell migration assay, 2 × 10^4^ cells were seeded in the upper chamber of a transwell insert (8 μm pore size; BD Biosciences, San Jose, CA, USA) in serum-free medium. The transwell inserts were placed in a 24-well plate. As for the invasion assay, prior to seeding, 4 × 10^4^ cells were treated with matrigel (Corning, USA) and incubated for 2 h. Complete culture medium was added to the lower chamber, and the cells were incubated for 24 h. After incubation, the cells in the upper chamber were removed using a cotton swab. The cells on the surface beneath the membrane were fixed with methanol, stained with hematoxylin, and then counted.

### Proliferation assay-EdU test

A549 or H1299 cells were transfected with CALU plasmid and siRNA for 48 hours. Subsequently, 1 × 10^5^ cells were seeded into 96-well plates and cultured until reaching the desired growth stage. 5-ethynyl-2’-deoxyuridine (EdU) (Beyotime, Shanghai, China) solution (reagent A) was diluted with cell culture medium at a ratio of 1:1000 to prepare a suitable amount of 50 μM working solution. The cells were then incubated in the presence of the EdU medium for 2 h. Following this, the cells were fixed with 4% paraformaldehyde, permeabilized with 0.5% Triton X-100 solution, and subjected to staining according to the instructions provided with the reagent kit. Finally, images were captured and analyzed. Proliferating cells were visualized as red fluorescence.

### Proliferation assay-colony formation

To assess the growth of monolayer cultures, 2 × 10^3^ cells/well were seeded in 6-well plates following transfection with the CALU overexpression plasmid and siRNA3. The medium was changed every three days. After a 14-day incubation period, the cells were fixed with 4% paraformaldehyde and stained with hematoxylin staining solution, (Solarbio, Beijing, China), and mages were captured.

### Enzyme-linked immunosorbent assays (ELISA)

A549 cells were transfected with CALU overexpression plasmids or siRNAs, and after 48 h, the cell culture supernatant was harvested. The supernatant was subjected to centrifugation and analyzed using the CALU ELISA kit (ResearchCloud, Shandong, China) following the provided instructions. Finally, the optical density (OD) values of each group were measured using a microplate reader.

### Lung adenocarcinoma organoid culture

After the dissection of lung adenocarcinoma organoid is completed, rinse them with organoid basic culture medium (Biogenous, Suzhou, China). Digest tissue fragments in a 15mL centrifuge tube using 10mL of tumor tissue digestion solution at 37 °C. The incubation time can range from 15 min to 45 min. After that, add fetal bovine serum to the tissue digestion mixture to achieve a final concentration of 2%, and filter the mixture through a 100 μm cell filter. Remove the supernatant, resuspend the particles in the basic culture medium, and prepare a seeding plate. Place the plate in a humidified incubator at 37 °C with 5% CO2. Change the culture medium every 3–4 days.

### Lung adenocarcinoma organoid validate by immunofluorescence

Resuspend the organoids in 1 mL of 4% paraformaldehyde, place the test tube on its side, and gently shake it for incubation in a fixed solution for 45 min. Following this, resuspend the organs in 1 mL of phosphate buffer saline (PBS) and proceed with antigen retrieval, permeabilization, and blocking steps. Remove the permeabilization/blocking solution and perform 3 washes with immunofluorescence (IF) buffer. Prepare a mixture of primary antibodies (diluted in IF buffer at a ratio of 1:400) and add 0.5 mL to the organoids. Mouse monoclonal anti-Napsin A was purchased from Proteintech (PTG, USA). Gently shake overnight at 4 °C. The dilution for the secondary antibody is 1:1000. Remove the first antibody solution and wash the organoids three times with IF buffer. Add 0.5 mL of the secondary antibody mixture (IF buffer + 10% goat serum + second antibody). Incubate at 4 °C for 24 h. Introduce DAPI directly to the organoids and gently shake them at room temperature in the dark for 15–20 min. Remove the 4’,6-diamidino-2-phenylindole fluorescent stain (DAPI) (Thermo Fisher, USA) and secondary antibody solution, transfer the organoids onto a glass-bottom chamber slide (8-well slide or confocal dish), and immediately proceed with imaging using a confocal microscope.

### Immunohistochemistry (IHC)

The tissue samples were fixed in 4% paraformaldehyde and subsequently embedded in paraffin. For immunohistochemistry, rabbit polyclonal anti-calumenin antibody was used as the primary antibody. Tissue sections of 3 μm thickness were prepared. The sections were baked at 55℃ for 2 h, followed by antigen retrieval using citrate buffer. Blocking was performed with goat serum, and then the sections were incubated with the calumenin antibody. Afterward, the tissue sections were incubated with a secondary antibody and visualized using diaminobenzidine (DAB) (Zhongshanjinqiao, Beijing, China) staining. Counter staining was done with hematoxylin, and then the sections were dehydrated, cleared, and mounted with neutral mounting medium. Images were observed and captured under a microscope.

### Statistical analysis

All data are reported as mean ± standard error of the mean (SEM). Statistical analysis was conducted using Prism 8 software. Comparisons between two groups were assessed using a t-test. A *p*-value < 0.05 was considered statistically significant.

## Results

### The upregulation of CALU in LUAD is related to an unfavorable prognosis

**Fig. 1:**
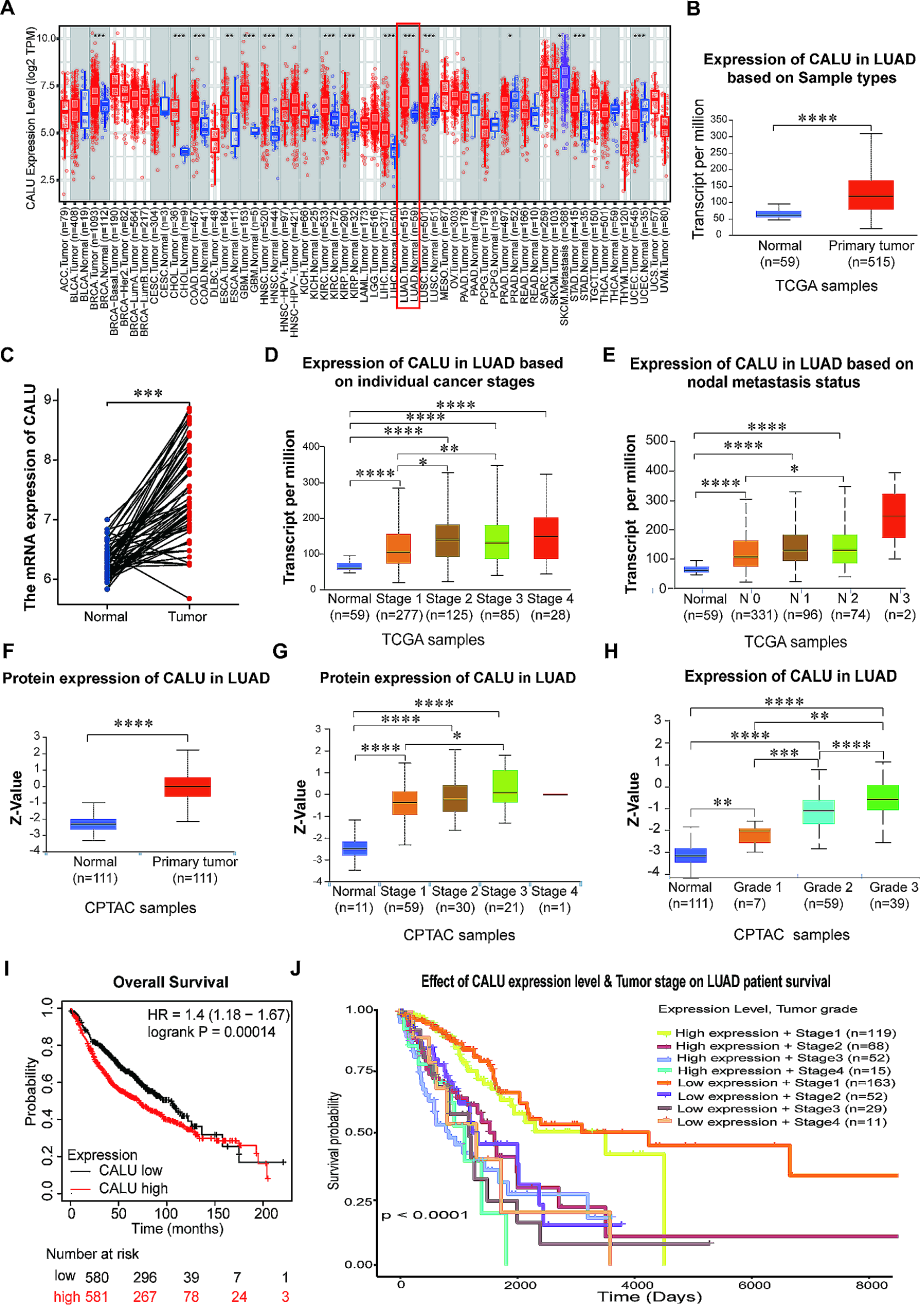
High expression of CALU in LUAD patients is associated with poor prognosis. **A-B** CALU RNA expression differences between normal and tumor tissues in pan-cancer was analyzed using data from the TIMER website (**A**) and UALCAN website (**B**). **C** The RNA expression differences between LUAD patients and paired normal tissues. **D** CALU RNA expression differences between normal(*n* = 59) and tumor tissues in LUAD based on cancer stages (Stage1, *n* = 277. Stage2, *n* = 125. Stage3, *n* = 85. Stage4, *n* = 28) from the TCGA database. **E** CALU RNA expression differences between normal(*n* = 59) and tumor tissues in LUAD based on nodal metastasis status (N0, *n* = 331. N1, *n* = 96.N2, *n* = 74.N3, *n* = 2) from the TCGA database. **F** CALU protein expression differences between normal(*n* = 111) and tumor(*n* = 111) tissues in LUAD from the CPTAC database. **G** CALU protein expression differences between normal(*n* = 11) and tumor tissues in LUAD based on cancer stages (Stage1, *n* = 59. Stage2, *n* = 30.Stage3, *n* = 21.Stage4, *n* = 1) from the CPTAC database. **H** CALU protein expression differences between normal(*n* = 111) and tumor tissues in LUAD based on grade (Grade1, *n* = 7. Grade2, *n* = 59.Grade3, *n* = 39) from the CPTAC database. **I** Kaplan–Meier curves of overall survival of LUAD patients with high versus low expressions of CALU (Logrank *P* = 0.00014). **J** Relationship between high or low expression of CALU and LUAD patient survival based on different pathological stages. *p<0.05.**p<0.01.***p<0.001.****p<0.0001

To explore the expression pattern of CALU across multiple cancers, we utilized the Tumor IMmune Estimation Resource(TIMER) (https://cistrome.shinyapps.io/timer/) (Fig. 1A) and SangerBox (http://sangerbox.com/) (Fig S1A) to discover upregulation of CALU in various malignancies, including LUAD, Breast Cancer (BRCA), Head and Neck Squamous Cancer (HNSC). The differential expression of CALU was analyzed in 515 tumor tissues and 59 normal tissues using The Cancer Genome Atlas (TCGA), revealing significant differences between lung tumor tissues and their corresponding normal counterparts. Notably, we observed that RNA expression of CALU was increased in LUAD tissues (Fig. 1B). By comparing CALU levels in LUAD and paired normal tissues using Log2(TPM + 1) quantification, we observed up-regulation of CALU in the majority of LUAD samples (Fig. 1C). Furthermore, the TCGA data revealed a correlation between high CALU expression and advanced stages of LUAD, indicating that higher expression levels corresponded to increased stages (Fig. 1D). Meanwhile, the TCGA database indicated a connection between high CALU expression and lymph node metastasis in LUAD, where higher CALU expression levels were associated with increased metastatic involvement (Fig. 1E).

Additionally, protein expression analysis was conducted using the Clinical Proteome Tumor Analysis Consortium (CPTAC) database confirmed the elevated expression of CALU in primary LUAD tumor samples (Fig. 1F). The CPTAC data similarly revealed a correlation between high CALU protein expression and advanced stages (Fig. 1G) or advanced grades (Fig. 1H) in LUAD, indicating that higher protein expression levels corresponded to increased cancer progression. We analyzed the relationship between CALU expression and the overall survival of LUAD patients using KM plot website (http://kmplot.com) (Fig. 1I), Gepia (http://gepia2.cancer-pku.cn/#index) (Fig S1B) and UALCAN (https://ualcan.path.uab.edu) (Fig S1C). We found that high expression of CALU was associated with poor prognosis in LUAD. Meanwhile, we analyzed the relationship between CALU expression and post progression survival, relapse free survival of LUAD using KM plot website, the results demonstrated a significant association between high CALU expression and poor prognosis in LUAD (Fig S1D-S1E). Finally, we downloaded the LUAD data from the TCGA database and compared the effects of high and low expression of CALU based on pathological stages on LUAD patient survival (Fig. 1J, Fig S1F). It showed that the lower pathological stage and the lower CALU expression was associated with the longer survival of LUAD patients; conversely, the higher pathological stage, the higher CALU expression, and the shorter survival period.

In summary, these comprehensive results highlight the involvement of CALU expression in cancer, particularly its upregulation of mRNA and protein in LUAD, and its association with poor prognosis, staging, and lymph node metastasis. These findings underscore the importance of further investigating the role of CALU in LUAD.

### CALU is upregulated in LUAD tissues and LUAD cell lines

**Fig. 2:**
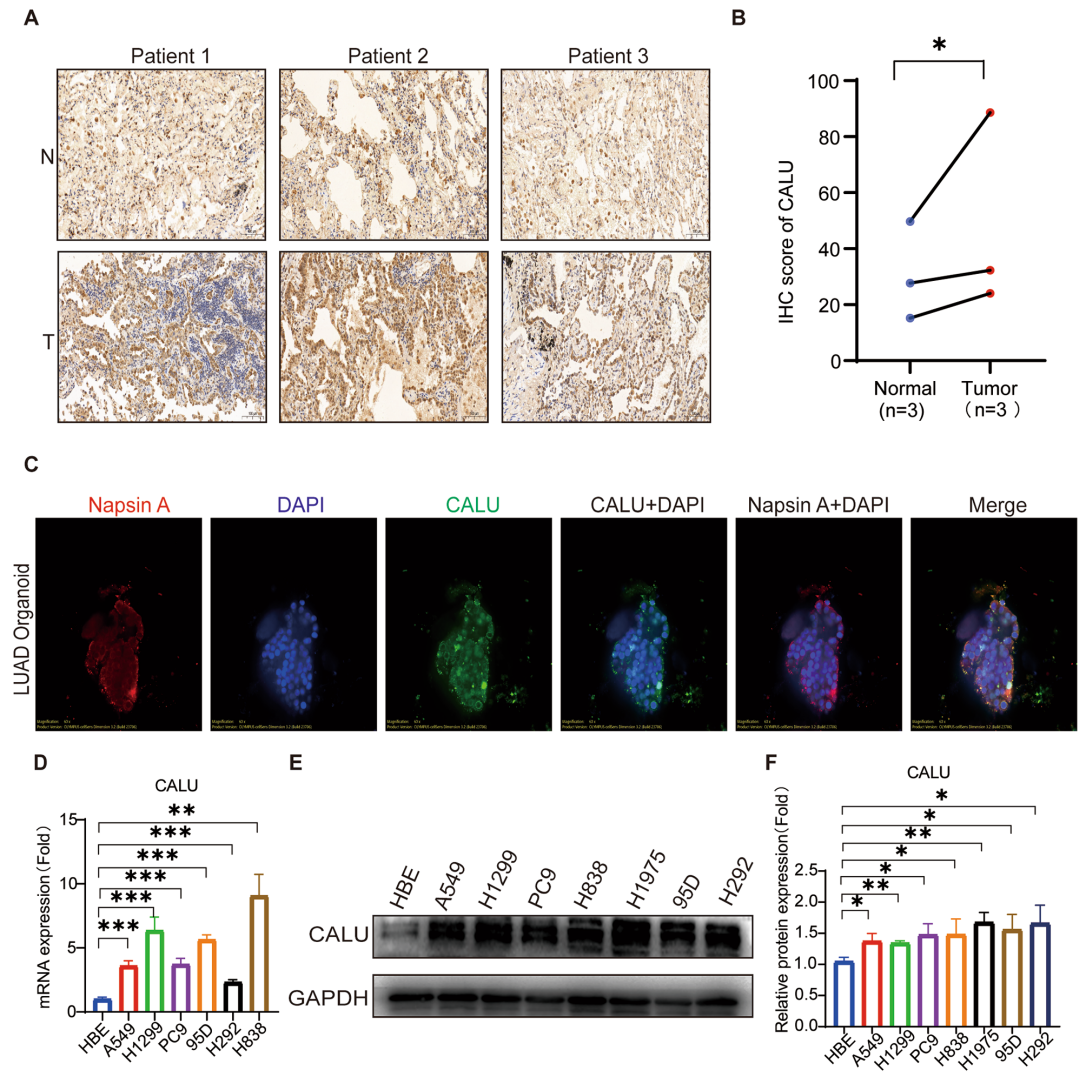
CALU is upregulated in LUAD tissues and LUAD cell lines. **A** Representative image of IHC staining for CALU in LUAD patient’s tumor and adjacent tissues, and relative proteins were be quantified. P, patient. N, normal. T, tumor. Scale bars = 100 mm. **B** Tumor, *n* = 3. Normal, *n* = 3.*p<0.05. **C** Representative images of IF staining for CALU in LUAD organoids. Red, Napsin A. Blue, Dapi. Green, CALU. **D-F** qRT-PCR (**D**) and western blotting assays (**E**) were performed to determine CALU expression in HBE and LUAD cell lines, in which CALU was quantified based on GAPDH (**F**). *p<0.05.**p<0.01, ***p<0.001

To further examine CALU expression, we collected surgically resected LUAD tissues and adjacent non-cancerous tissues. Immunohistochemical staining demonstrated higher CALU expression in LUAD tissues compared to the adjacent tissues (Fig. 2A and B). Additionally, given that 3D organoids faithfully preserve the genetic and phenotypic characteristics of their parent tumor, patient-derived organoids (PDOs) have recently emerged as robust preclinical models. In this study, our initial findings revealed that calumenin was highly expressed in LUAD organoids by using immunofluorescence. We verified their identity using Napsin A as a specific marker for LUAD (Fig. 2C). In our study, we employed qRT-PCR and western blotting techniques to investigate the mRNA and protein expression of CALU in HBE cells as well as various LUAD cell lines, including A549, H1299, H1975, PC9, H838, H292 and 95D. Our findings exhibited elevated levels of CALU mRNA (Fig. 2D) and protein (Fig. 2E and F) in different LUAD cell lines compared to HBE cells.

### CALU enhances the proliferation and migration of A549 and H1299 cells

**Fig. 3:**
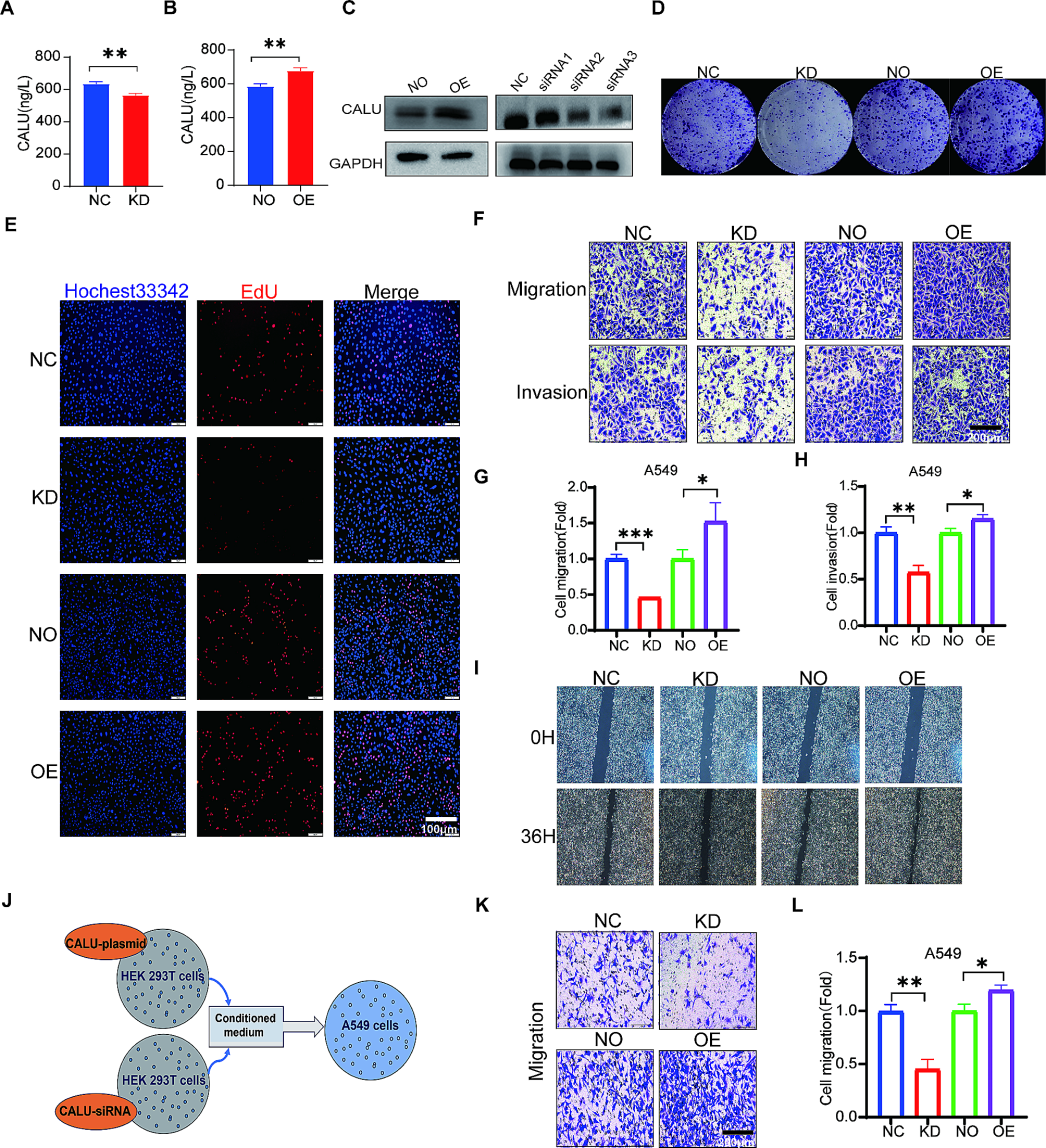
CALU knockdown inhibits proliferation and migration in A549 cells. **A** Detection of CALU content in cell supernatant after transfection with CALU siRNA using ELISA. **p <0.01. **B** Detection of CALU content in cell supernatant after transfection with CALU overexpression plasmid using ELISA. **p <0.01.**C-I** CALU was overexpressed or knocked down in A549 cells, which were subsequently subjected to western blotting analysis (**C**). The effects of CALU overexpression or knockdown on cell proliferation were assessed using colony formation assays (**D**) and EdU assays (**E**). Bar = 100 μm. Transwell assays (**F**) were conducted to measure cell metastasis, and quantitative analysis of migration and invasion data from three independent experiments were also conducted (**G-H**). Bar = 200 μm. Wound healing assays (**I**) were also conducted to measure cell metastasis. **J-L** A549 cells were cultured using supernatant from HEK293T cells transfected with CALU siRNA or overexpressing plasmids (**J**) followed by migration assays (**K**), and quantitative analysis of migration data from three independent experiments were also conducted (**L**). Bar = 200 μm .*p<0.05.**p<0.01. ***p<0.001

As CALU is known to be a secretory protein with intracellular and extracellular distribution, we employed an ELISA assay kit to measure CALU levels in the supernatant of A549 cells transfected with CALU overexpression plasmids or CALU-specific siRNA. The results showed similar CALU levels in the supernatant, consistent with the cellular expression patterns (Fig. 3A and B). We selected LUAD cell lines A549 and H1299 for subsequent cellular phenotype experiments. We validated the transfection efficiency of CALU through western blotting in A549 (Fig. 3C) and H1299 cells (Fig S2A-S2B). We selected siRNA3 to knock down CALU for phenotypic experiments. To investigate the impact of CALU on cell proliferation in LUAD, we performed colony formation assays and EdU assays. Both experiments revealed that suppressing CALU expression repressed cell proliferation, whereas overexpressing CALU promoted the proliferation of LUAD cells. (Figure 3D and E, Fig S2C-S2D). Transwell assays demonstrated that silencing CALU inhibited the migration and invasion of LUAD cells, while overexpressing CALU further enhanced their migration and invasion abilities (Fig. 3F–H, Fig S2E-S2G). We conducted wound healing assays to find that silencing CALU inhibited the wound healing while overexpressing CALU further enhanced their wound healing (Fig. 3I, Fig S2H). In order to verify that CALU can secrete and exert its effects outside the cell, we added conditioned medium from HEK293T cells overexpressing and silencing CALU to A549 cells for 24 h to observe cell migration (Fig. 3J). We also found that the migration ability of A549 cells was affected. Knocking down CALU in the supernatant inhibited A549 cell migration, while overexpressing CALU in the supernatant promoted A549 cell migration (Fig. 3K and L).

### RNA sequencing analysis of CALU knockdown in A549 cells

**Fig. 4:**
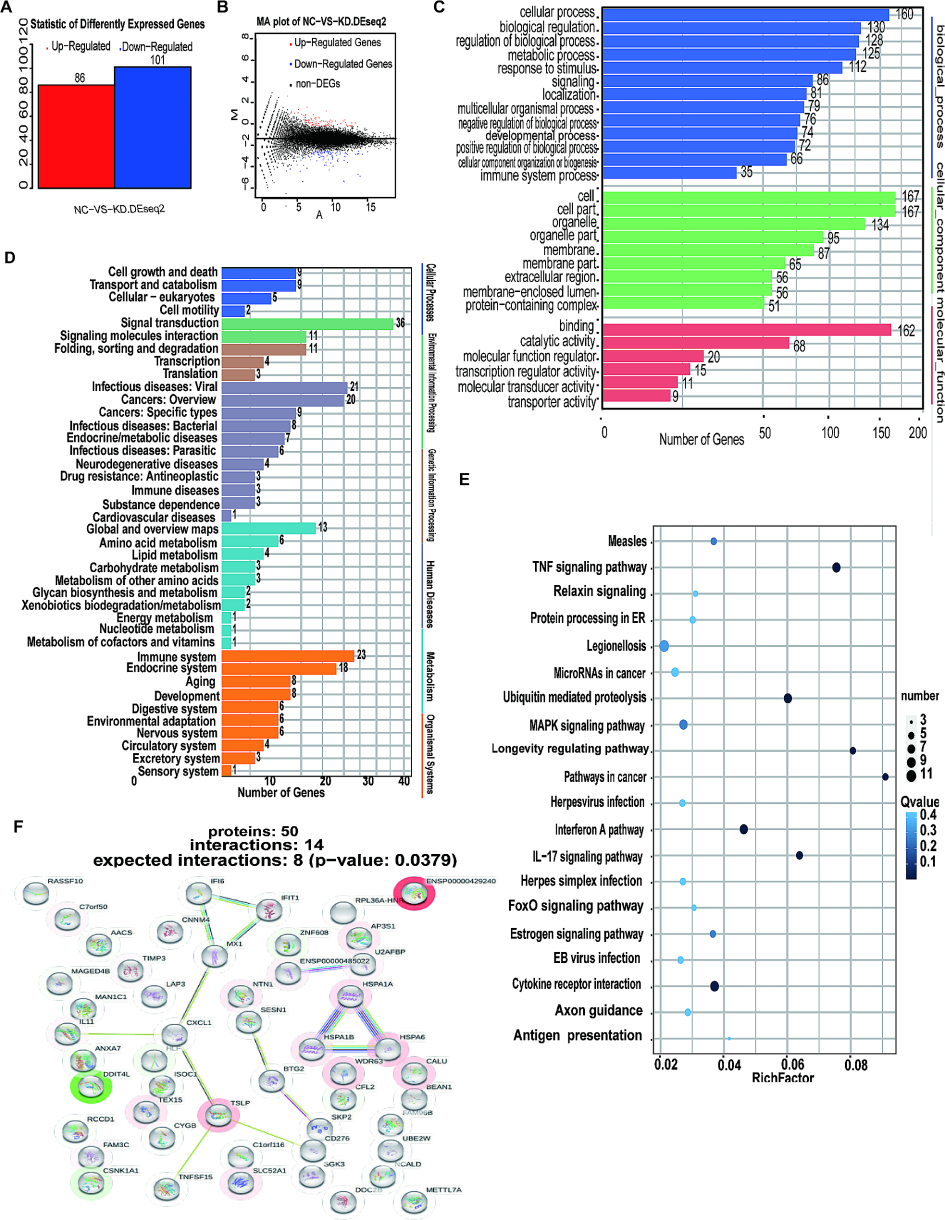
Transcriptomics of LUAD cells reveals the mechanism of CALU knockdown in cancer suppression. **A** The bar graph presents the genes significantly affected by CALU knockdown. DEGs were selected based on the criteria of |log2(FoldChange)|>1 and q-value < 0.001. **B** Volcano diagram of DEGs. Red stands for up-regulated genes and blue stands for down-regulated genes. **C-D** GO analysis based on DEGs. **E-F** KEGG analysis (**E**) and PPI analysis(**F**) predicts 50 possible interacting proteins based on DEGs.

Based on our bioinformatics analysis, we aimed to investigate the specific mechanisms through which CALU promotes LUAD progression. we performed RNA sequencing analysis in A549 cells. Gene expression is known to exhibit specificity in both temporal and spatial contexts, and genes with significantly different expression levels under distinct experimental conditions are referred to as differentially expressed genes (DEGs). Utilizing the PossionDis algorithm for differential gene detection, we compared CALU knockdown with lung adenocarcinoma cells. DEGs were selected based on the criteria of |log2(FoldChange)| > 1 and q-value < 0.001. Through this screening process, we identified a total of 187 DEGs, comprising 86 upregulated genes and 101 downregulated genes following CALU knockdown (Fig. 4A and B).

To gain a better understanding of the functional classification and enrichment analysis of the identified DEGs, we performed a GO enrichment analysis to categorize the DEGs into biological processes, molecular functions, and cellular components. The biological process category included terms such as cellular processes, biological regulation, and cellular component comprised of organelle and extracellular region, while the molecular function category consisted of binding, catalytic activity, and molecular function regulation (Fig. 4C).

In addition, we conducted KEGG analysis to identify significant pathways associated with the DEGs and to elucidate the key biochemical metabolic and signaling pathways involved. The results were presented as pathway classifications, with each category representing the number of DEGs involved. These pathway categories included Cellular Processes (cell growth and death, transport and catabolism), Environmental Information Processing (signal transduction, signaling molecules and interaction), Genetic Information Processing (folding, sorting and degradation, transcription), Human Diseases (infectious diseases: viral and cancers), Metabolism (amino acid metabolism, lipid metabolism), and organismal systems (immune system, endocrine system, aging) (Fig. 4D). The bubble chart showed that DEGs were mainly enriched in pathways in cancer, tumor necrosis factor (TNF) signaling pathway, interferon A pathway, ubiquitin mediated proteolysis and interleukin17 (IL17) signaling pathway (Fig. 4E).

To investigate the protein-protein interactions among the DEGs, we utilized the STRING database to construct an interaction network by using BLAST software for protein-protein interaction (PPI) analysis. This allowed us to generate a protein interaction network diagram for a comprehensive overview of the protein interactions involved, and PPI diagram showed that the protein network related to CALU is mainly composed of inflammatory and immune related proteins, such as Heat Shock Protein A (HSPA) family, TNF, Thymic stromal lymphopoietin (TSLP), IL, C-X-C motif Chemokine Ligand (CXCL) (Fig. 4F).

### GSEA based on all the differentially expressed genes obtained from the transcriptomics

GSEA was performed on the entire set of differentially expressed genes obtained from the transcriptomics. Enrichment maps of various pathways were generated by calculating the enrichment score (ES) and estimating the significance level of the ES. For the analysis results, it is generally considered that pathways with |NES| > 1, NOM p-val < 0.05, and FDR q-val < 0.25 are significantly enriched.

**Fig. 5:**
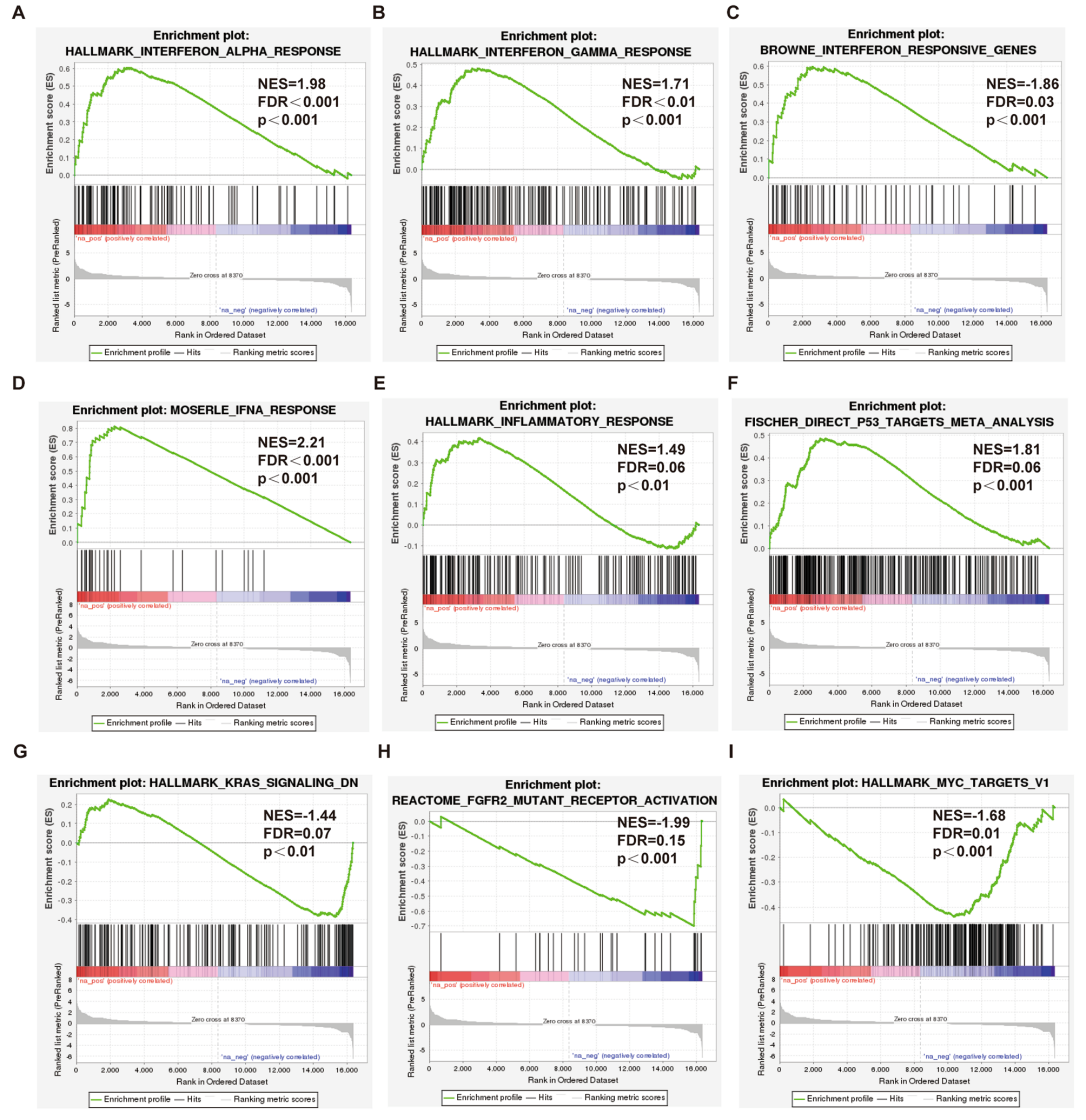
GSEA analysis based on all differentially expressed genes. **A-D** Enrichment plot showed interferon alpha (**A**) and interferon gamma (**B**) as hallmark, interferon responsive genes(**C**) and IFNA response(**D**) were activated. **E-F** Enrichment plot showed inflammatory response as hallmark(**E**) and p53 targets based on Meta analysis (**F**) were activated. **G-I** Enrichment plot showed KRAS signaling(**G**), FGFR2 mutant receptor activation (**H**) and MYC targets as hallmark (**I**) were inhibited

The analysis revealed that the interferon response, interferon A (IFNA) and interferon gamma response pathways were upregulated in the CALU knockdown group (Fig. 5A–D). Additionally, inflammatory signals (Fig. 5E) and the p53 pathway (Fig. 5F) were activated. However, signals associated with tumor suppressor pathways, such as V-Ki-ras2 Kirsten rat sarcoma viral oncogene homolog (KRAS) (Fig. 5G), Fibroblast Growth Factor Receptor 2 (FGFR2) (Fig. 5H), MYC (Fig. 5I), were notably inhibited in CALU knockdown group.

### IPA based on the significantly differentially expressed genes obtained from the transcriptomics for exploring potential pathways that may be affected

**Fig. 6:**
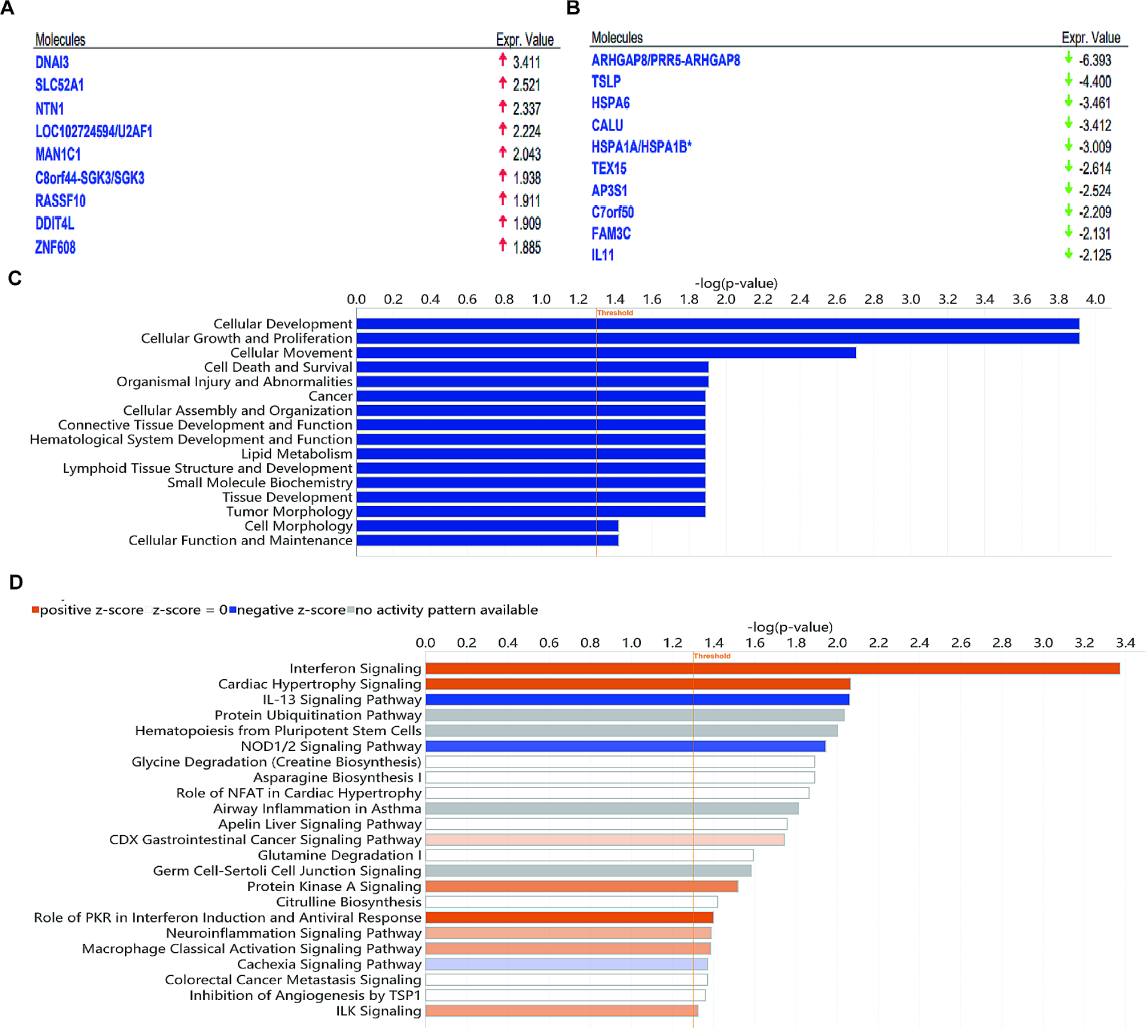
IPA based on the DEGs obtained from the transcriptomics for exploring potential anti-cancer mechanism. **A-B** Top ten genes significantly upregulated(**A**) and downregulated (**B**) after knocking down CALU in A549 cells. **C** Enrichment analysis of genes associated with diseases and functions. **D** Enrichment analysis of pathways based on DEGs. Orange represents pathway was significantly activation and blue represents pathway was significantly inhibition based on z-score

To further investigate the mechanism of CALU in LUAD progression, we conducted IPA analysis on the DEGs. After knocking down CALU in A549 cells, the significantly upregulated genes included DNAI3, SLC52A1, NTN1, U2AF1, MAN1C1, SGK3, RASSF10, DDIT4L, and ZNF608(Fig. 6A). However, the significantly downregulated genes included ARHGAP8, TSLP, HSPA6, HSPA1A, TEX15, AP3S1, C7orf50, FAM3C, and IL11(Fig. 6B). Enrichment analysis revealed that these genes were associated with various diseases and functions, including cancer, organismal injury and abnormalities, cellular development, cellular growth and proliferation, cellular movement, cell death and survival following CALU knockdown (Fig. 6C).

The pathway enrichment analysis indicated that several pathways were significantly enriched, including interferon signaling (Fig S3A), inhibition of angiogenesis by Thrombospondin-1 (TSP1) (Fig S3B), protein ubiquitination pathway (Fig S3C), role of tissue factor in cancer (Fig S4A and S4B), NOD1/2 signaling pathway (Fig S5), protein kinase A signaling (PKA) (Fig S6), IL-13 signaling pathway and WNT/β-catenin signaling. According to the Z-value results, interferon signaling, role of tissue factor in cancer, and protein kinase A signaling were significantly activated, while WNT/β-catenin signaling was inhibited (Fig. 6D).

**Fig. 7:**
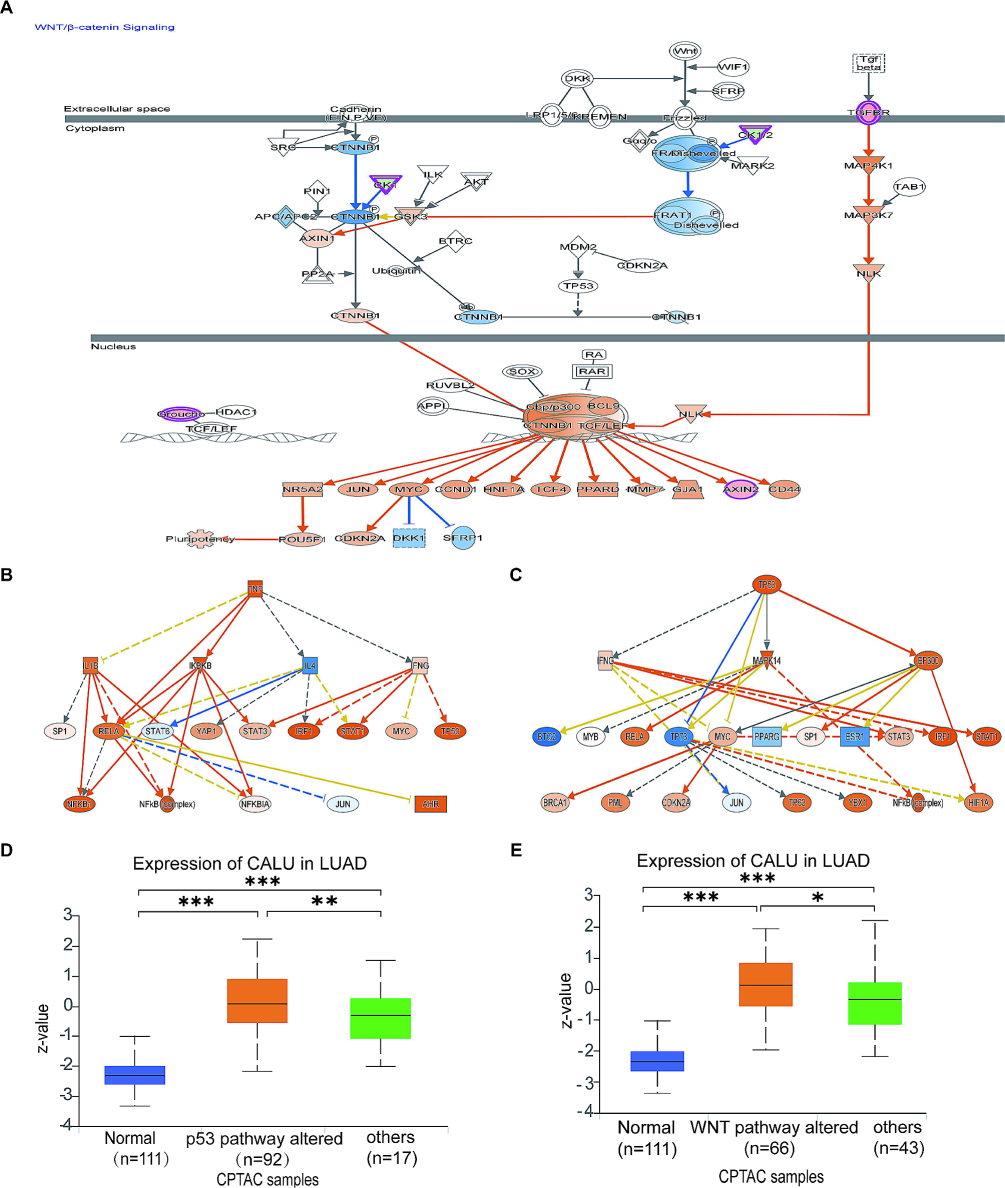
CALU knockdown leads to inhibition of the WNT signaling pathway and activation of p53 and TNF signals. **A** Important molecules in WNT/β-catenin signaling pathway and their upstream and downstream relationships based on IPA. **B-C** Downstream molecules affected by TNF(**B**) and TP53(**C**) activation based on IPA.**D-E** Expression level of CALU in LUAD is higher with p53 pathway altered (**D**) and WNT pathway altered (**E**) from CPTAC samples. *p<0.05.**p<0.01.***p<0.001

WNT/ β- catenin signaling plays an important role in the progression of various cancers, and this figure shows the important molecules involved in the WNT pathway (Fig. 7A). Moreover, tumor protein P53 (TP53), TNF, hematopoietic growth factor (HGF), vascular endothelial growth factor (VEGF), and casp1-casp5 were identified as vital upstream regulators, with TNF (Fig. 7B) and TP53 (Fig. 7C) showing significant activation. Furthermore, based on the query in the CPTAC sample bank, CALU was found to be highly expressed in the abnormal P53 pathway (Fig. 7D) associated with LUAD, as well as in the activated pathway of WNT (Fig. 7E).

Taken together, we found that the anti-cancer mechanism of CALU knockdown might be related to the inhibition of MYC and KRAS signaling pathways but the activation of interferon signals, inflammatory signals and p53 pathways. High expression of CALU promotes the progression of LUAD by inhibiting a variety of cancer-inhibiting pathways and activating classical cancer-promoting pathways, therefore, CALU may be an important therapeutic target for LUAD.

## Discussion

CALU exhibits the highest expression in the heart, placenta, and skeletal muscle, while its levels are lower in lungs, kidneys, and pancreas [24]. Study shows that calumenin has an important regulatory role in membrane proteins [25, 26]. CALU promotes vascular calcification by reducing gamma-carboxylation and inhibiting the activation of matrix Gla protein [27, 28]. Furthermore, calumenin interacts with and inhibits the sarcoplasmic reticulum (SR) ryanodine receptor and the cardiac Ca2 + transporting ATPase. Differential regulation of the CALU transcript has been observed in various malignant diseases and malignant cells, indicating its potential as a prognostic marker. Studies on gliomas [23] have highlighted that CALU related genes are closely associated with the epithelial-mesenchymal transition (EMT) phenotype. In mucosal melanoma cells, overexpression of miR-let-7b or miR-let-7c [29] inhibits cell growth, migration, invasion, and metastasis while inducing apoptosis and cell cycle arrest both in vitro and in vivo. These effects are achieved through targeting CALU and inhibiting phosphorylated extracellular signal-regulated kinase (ERK) signaling. Additionally, CALU has been implicated in bladder cancer prognosis [30], where it is involved in tumor microenvironment remodeling, gene mutations, and iron-dependent cell death. The spliceosome calumenin-15, which possesses nuclear localization signals, has been found to play a significant role in cell migration and tumor metastasis [31]. However, studies demonstrated lower expression of CALU in certain tumors, such as downregulation of the metastasis-related protein calmodulin precursor in head and neck cancer cell lines [32] and hepatocellular carcinoma cells [33]. In lung squamous cell carcinoma, the CALU transcript was found to be downregulated compared to normal lung tissue.

Calumenin has emerged as a potential marker and effective therapeutic target for LUAD. However, there is limited research on CALU in malignant tumors, and the understanding of its mechanisms in cancer is still nascent. In this study, we have highlighted the following key findings:1. CALU is upregulated in LUAD and is associated with poor prognosis. 2. CALU promotes migration and proliferation of LUAD cells.3. Inhibition of CALU can exert anti-cancer effects by activating pathways such as TNF, IFN, TP53, and the tissue factor pathway, while inhibiting the WNT pathway. These findings provide compelling evidence that CALU plays a critical role in the progression of lung cancer, particularly LUAD.

In our present study, we performed GO analysis, KEGG analysis, GSEA and IPA based on transcriptomics by comparing CALU knockdown with A549 cells. Although no single pathway showed a high concentration of suggestions, considering the multifaceted effects of CALU in LUAD, it is suggested that CALU, as an upstream regulator, can influence multiple pathways and thus impact the progression of this disease. GSEA analysis revealed that knockdown of CALU in A549 cells significantly inhibited MYC and KRAS signals while activating interferon signals, inflammatory signals, and p53 pathways. These results were statistically significant. Furthermore, IPA analysis provided additional insights, indicating that the interferon and inflammatory pathways were prominently activated upon CALU knockdown in A549 cells.

Interferons (IFNs) are a class of proinflammatory cytokines with anticancer effects by directly inhibiting the growth of malignant cells and stimulating immune responses in tumor progression, malignant transformation and response to therapy [34, 35]. The enrichment analysis of DEGs following CALU knockdown in A549 cells highlighted the strong activation of the interferon pathway. This suggests that CALU may be secreted into the tumor microenvironment through exocrine pathways, inhibition of CALU enhances anti-tumor immunity and inhibiting tumor progression. In cancers, inhibitors of IL-13 could enhance anti-tumor defenses, thus serving as potential cancer immune therapeutics [36]. Our findings highlighted the strong inhibition of the IL-13 pathway after silencing CALU.

Our analysis also showed that anti-tumor effect of CALU is related to the activation of the ubiquitin system and inhibition of the Nucleotide-Binding Oligomerization Domain 1/2 (NOD1/2) and PKA pathways. In tumor cells, both ubiquitination and deubiquitination processes play a role in regulating the metabolic reprogramming of cancer cells [37]. Ubiquitination has been reported to be involved in the metabolic reprogramming of cancer cells through the targeting of various molecules, including AKT serine/threonine kinase (AKT) [38], phosphatase and tensin homolog (PTEN) [39], KRAS [40], epidermal growth factor receptor (EGFR) [41], hypoxia-inducible factor (HIF) [42], c-Myc [43], p53 [44], nuclear factor erythroid 2-related factor 2 (NRF2) [45], mitogen-activated protein kinase (MAPK) [46], and ERK [47]. NOD1/2 pathway is involved in regulating the development of various tumors. In LUAD, a systematic exploration of pyroptosis related genes (PRGs) was conducted for prognosis prediction, including NOD1 [48]. Research has shown that PKA regulates gene expression, cell survival, and migration [49, 50].

In addition, TSP-1, Integrin-linked kinase (ILK), Tissue factor (TF), WNT signaling pathways are significantly inhibited after CALU knockdown. TSP-1 is an exceptionally potent anti-angiogenic factor that impedes blood vessel growth by preventing endothelial cells from responding to various pro-angiogenic signals. A study [51] unveiled that intratumoral administration of monoclonal antibodies against TSP-1 effectively curtailed tumor growth and metastasis, accompanied by an increase in tumor-specific tumor-infiltrating lymphocytes and systemic immune responses. ILK, a serine/threonine kinase, has been implicated in the oncogenesis and progression of human cancers by activating signaling pathways that promote cell proliferation, migration, and EMT [52]. In addition to its role in hemostasis, TF expression by tumor cells contributes to various pathological processes, including thrombosis, metastasis, tumor growth, and tumor angiogenesis [53]. Inhibiting TF could potentially attenuate multiple pathological pathways associated with increased tumor growth and metastasis. The classical WNT signaling pathway is involved in EMT and promoting oncogene expression by inhibiting the phosphorylation of cytoplasmic β-catenin mediated by glycogen synthase kinase-3β (GSK3β), leading to its stabilization and nuclear translocation. Small molecule inhibitors that target crucial posttranslational modifications of WNT proteins have demonstrated effectiveness in reducing tumor growth and significantly decreasing the proliferative potential of lung cancer cells [54].

Currently, our study, in conjunction with bioinformatics analysis, has revealed the significant involvement of CALU in the progression of LUAD. Specifically, CALU appears to exert notable effects on the proliferation, migration and invasion of LUAD cells. However, it is important to note that our experiments have thus far been limited to in vitro cellular investigations, and we have not yet conducted animal experimental studies. Furthermore, our exploration of the underlying mechanisms is still in its early stages, necessitating further verification. Through such verification, we aim to establish a more robust experimental foundation for our conclusions and provide a scientific basis for the future development and clinical translation of small molecule compounds targeting CALU.

## Conclusions

In summary, our findings indicate that CALU is significantly upregulated in LUAD and contributes to the progression of this cancer by promoting the migration and proliferation. The expression pattern of CALU varies across different cancer types, suggesting its multifaceted and context-specific mechanism of action. Notably, we observed an opposite expression pattern of CALU in squamous cell carcinoma compared to adenocarcinoma, such as in lung squamous cell carcinoma and lung adenocarcinoma. This intriguing observation warrants further investigation into the specific mechanisms underlying the divergent roles of CALU. Understanding these mechanisms could facilitate the development of small molecule compounds targeting CALU for therapeutic interventions tailored to different cancer types, thereby offering a promising avenue for clinical translation. Moreover, our study provides a robust experimental foundation for a comprehensive and in-depth understanding of the molecular function of CALU.

### Electronic supplementary material

Below is the link to the electronic supplementary material.

**Supplementary Material 1: Fig S1.** High expression of CALU in LUAD patients is associated with poor prognosis. **Fig S2.** CALU knockdown inhibits proliferation and migration in H1299 cells. **Fig S3.** The signaling pathways which are activated after CALU knocking down. **Fig S4.** Roles of tissue factor in cancer. **Fig S5.** NOD1/2 signaling pathway. **Fig S6.** Protein kinase A signaling pathway

**Supplementary Material 2: Additional file 2:** RNA sequencing Analysis of CALU

Supplementary Material 3

## Data Availability

No datasets were generated or analysed during the current study.

## Abbreviations

AKT: AKT serine/threonine kinase
ATCC: American Type Culture Collection
BCA: Bicinchoninic acid
BRCA: Breast Cancer
BSA: Bovine Serum Albumin
CALU: Calumenin
CPTAC: Clinical Proteome Tumor Analysis Consortium
CREC: Ca 2+ -binding protein of 45 kDa, reticulocalbin, ER Ca 2+ -binding protein of 55 kDa, calumenin
CXCL: C-X-C motif Chemokine Ligand
DAB: Diaminobenzidine
DAPI: 4’,6-diamidino-2-phenylindole
DEGs: Differentially expressed genes
ECL: Enhanced chemiluminescence
EdU: 5-ethynyl-2’-deoxyuridine
EGFR: Epidermal growth factor receptor
ELISA: Enzyme-linked immunosorbent assay
EMT: Epithelial-mesenchymal transition
ERK: Extracellular signal-egulated kinase
ES: Enrichment score
FGFR2: Fibroblast Growth Factor Receptor 2
GO: Gene Ontology
GSEA: Gene Set Enrichment Analysis
GSK3β: Glycogen synthase kinase-3β
HBE: Human bronchial epithelial cells
HGF: Hematopoietic growth factor
HIF: Hypoxia-inducible factor
HNSC: Head and Neck Squamous Cancer
HSPA: Heat Shock Protein A
IF: Immunofluorescence
IFNA: Interferon A
IL17: Interleukin17
ILK: Integrin-linked kinase
IPA: Ingenuity Pathway Analysis
KEGG: Kyoto Encyclopedia of Genes and Genomes
KRAS: V-Ki-ras2 Kirsten rat sarcoma viral oncogene homolog
LUAD: Lung adenocarcinoma
MAPK: Mitogen-activated protein kinase
MMP-13: Matrix metalloproteinase13
NOD1/2: Nucleotide-Binding Oligomerization Domain 1/2
NRF2: Nuclear factor erythroid 2-related factor 2
NSCLC: Non-small cell lung cancer
PBS: Phosphate buffer saline
PDOs: Patient-derived organoids
PKA: Protein kinase A signaling
PMSF: Phenylmethylsulfonyl fluoride
PPI: Protein-protein interaction
PRGs: Pyroptosis related genes
PTEN: Phosphatase and tensin homolog
RIPA: Radio immunoprecipitation assay
SR: Sarcoplasmic reticulum
TCGA: The Cancer Genome Atlas
TF: Tissue factor
TIMER: Tumor IMmune Estimation Resource
TNF: Tumor necrosis factor
TP53: Tumor protein P53
TSLP: Thymic stromal lymphopoietin
TSP1: Thrombospondin-1
VEGF: vascular endothelial growth factor
