# Supplementary material for: CALU promotes lung adenocarcinoma progression by enhancing cell proliferation, migration and invasion
Source: Respir Res. 2024 Jul 5;25:267. doi: 10.1186/s12931-024-02901-3 (PMC11227236; doi:10.1186/s12931-024-02901-3)
Supplement: Supplementary file 1 — Supplementary Material 1: Fig S1. High expression of CALU in LUAD patients is associated with poor prognosis. Fig S2. CALU knockdown inhibits proliferation and migration in H1299 cells. Fig S3. The signaling pathways which are activated after CALU knocking down. Fig S4. Roles of tissue factor in cancer. Fig S5. NOD1/2 signaling pathway. Fig S6. Protein kinase A signaling pathway [file 12931_2024_2901_MOESM1_ESM.docx]

**
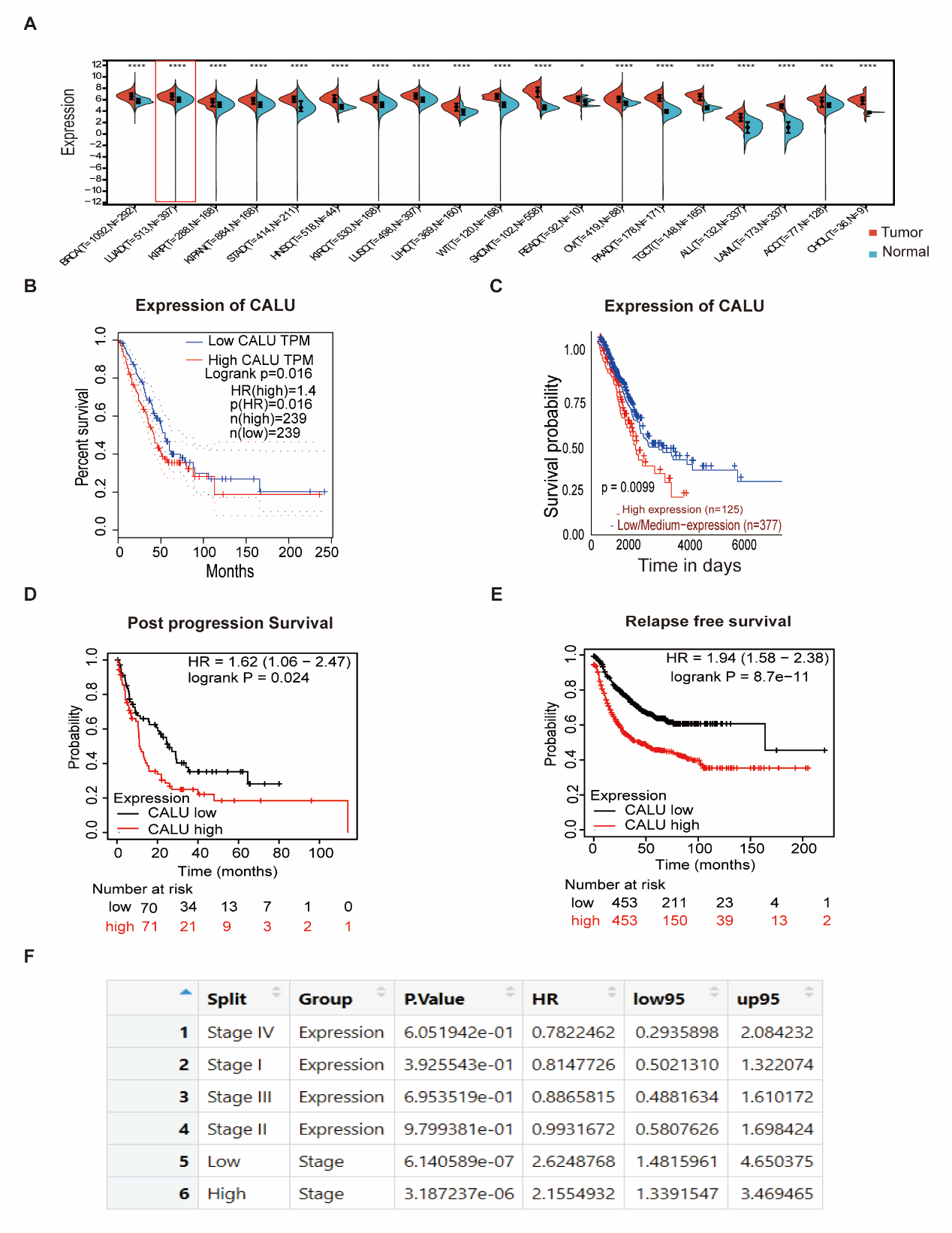
**

**Fig S1. High expression of CALU in LUAD patients is associated with poor prognosis.**

**A** CALU RNA expression differences between normal and tumor tissues in pan-cancer from SangerBox. **B** Curves of overall survival of LUAD patients with high versus low expressions of CALU (high, n=239. low, n=239. Logrank P=0.016) from Gepia website. **C** Curves of overall survival of LUAD patients with high versus low expressions of CALU (high, n=125.low/medium, n=377.P=0.0099) from Ualcan website. **D-E** Kaplan–Meier curves of survival of LUAD patients with high versus low expressions of CALU, including post progression survival(**D**) (Logrank P=0.024) and relapse free survival(**E**) (Logrank P=8.7e-11). F Statistical analysis based on CALU expression level or pathological stage on LUAD patient survival. * p＜0.05.**p＜0.01.***p＜0.001.****p＜0.0001.

**
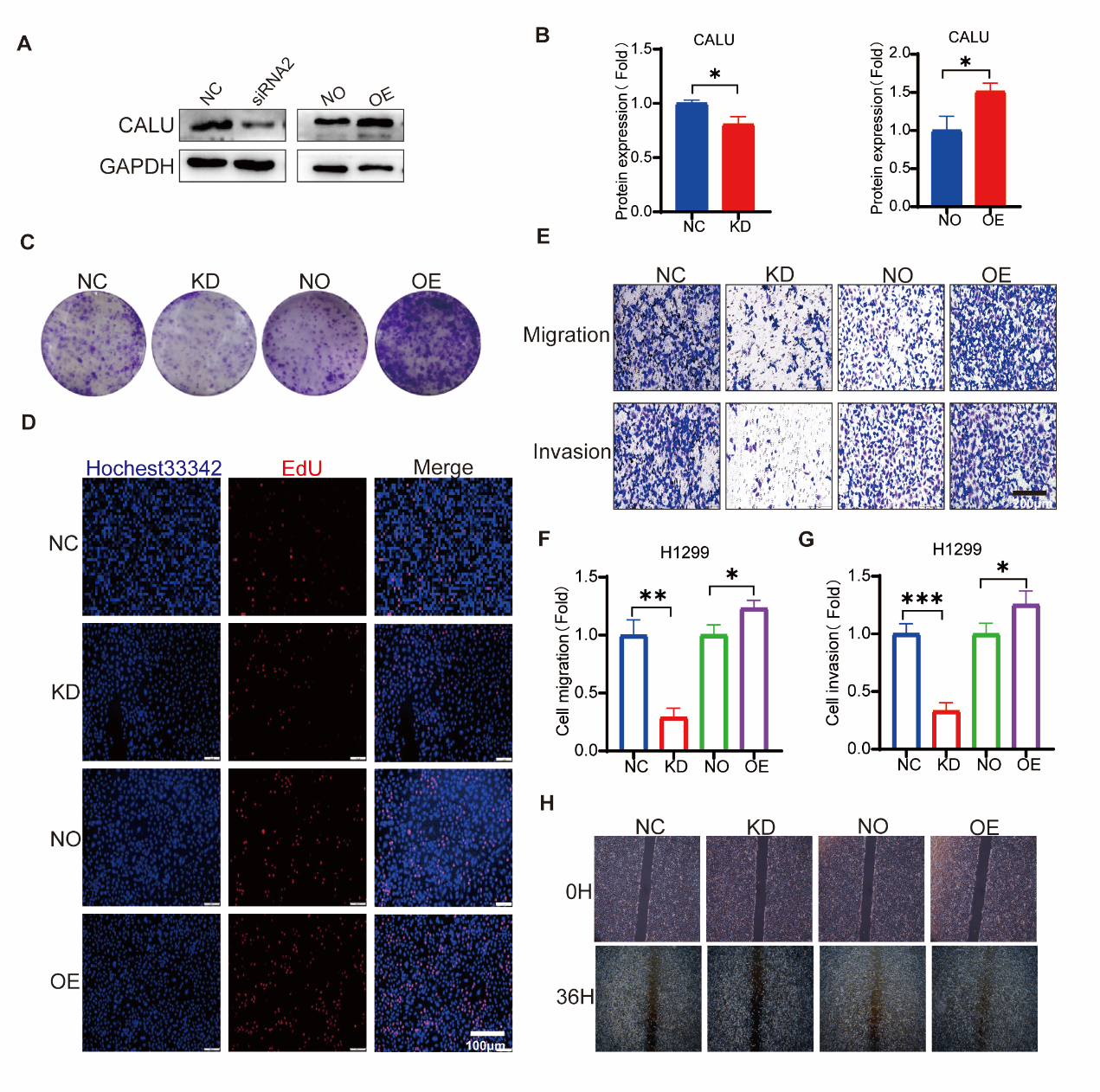
**

**Fig S2. CALU knockdown inhibits proliferation and migration in H1299 cells.**

**A-B** CALU was overexpressed or knocked down in H1299 cells, which were subsequently subjected to western blotting analysis(**A**) in which CALU was quantified based on GAPDH(**B**) *p＜0.05. **C-H** The effects of CALU overexpression or knockdown on cell proliferation were assessed using colony formation assays (**C**), EdU assays (**D**).Bar=100μm. Red represents proliferating cells. Transwell assays (**E**) were conducted to measure H1299 cell metastasis, and quantitative analysis of migration and invasion data from three independent experiments was also conducted (**F-G**).Bar=200 μm .*p＜0.05.**p＜0.01. ***p＜0.001. Wound healing assays (H) were also conducted to measure cell metastasis.

**
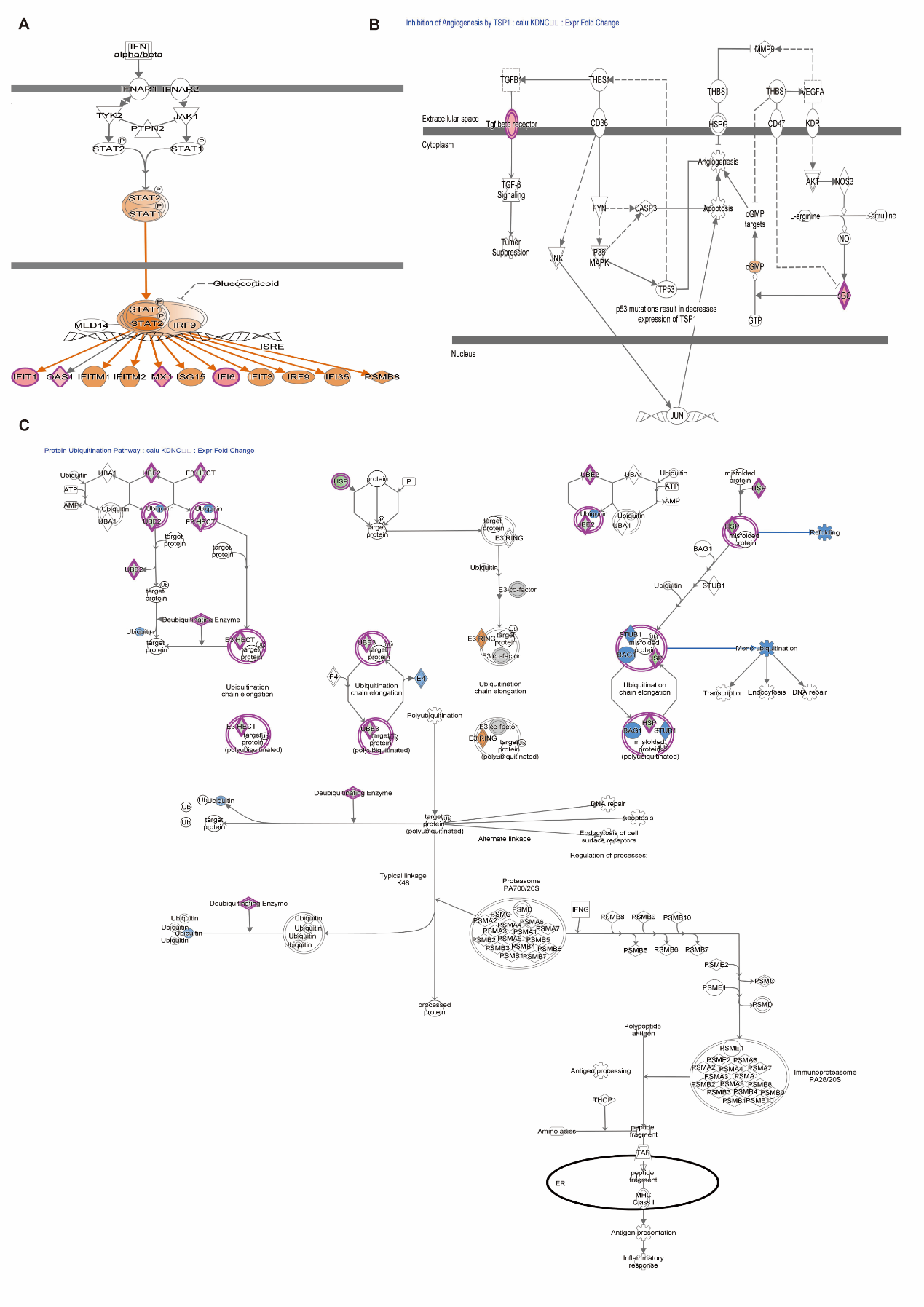
**

**Fig S3. The signaling pathways which are activated after CALU knocking down.**

**A-C** The IFN signaling pathway(**A**), inhibition of angiogenesis by TSP1(**B**) and protein ubiquitination pathway(**C**) are activated after knocking down CALU from IPA.


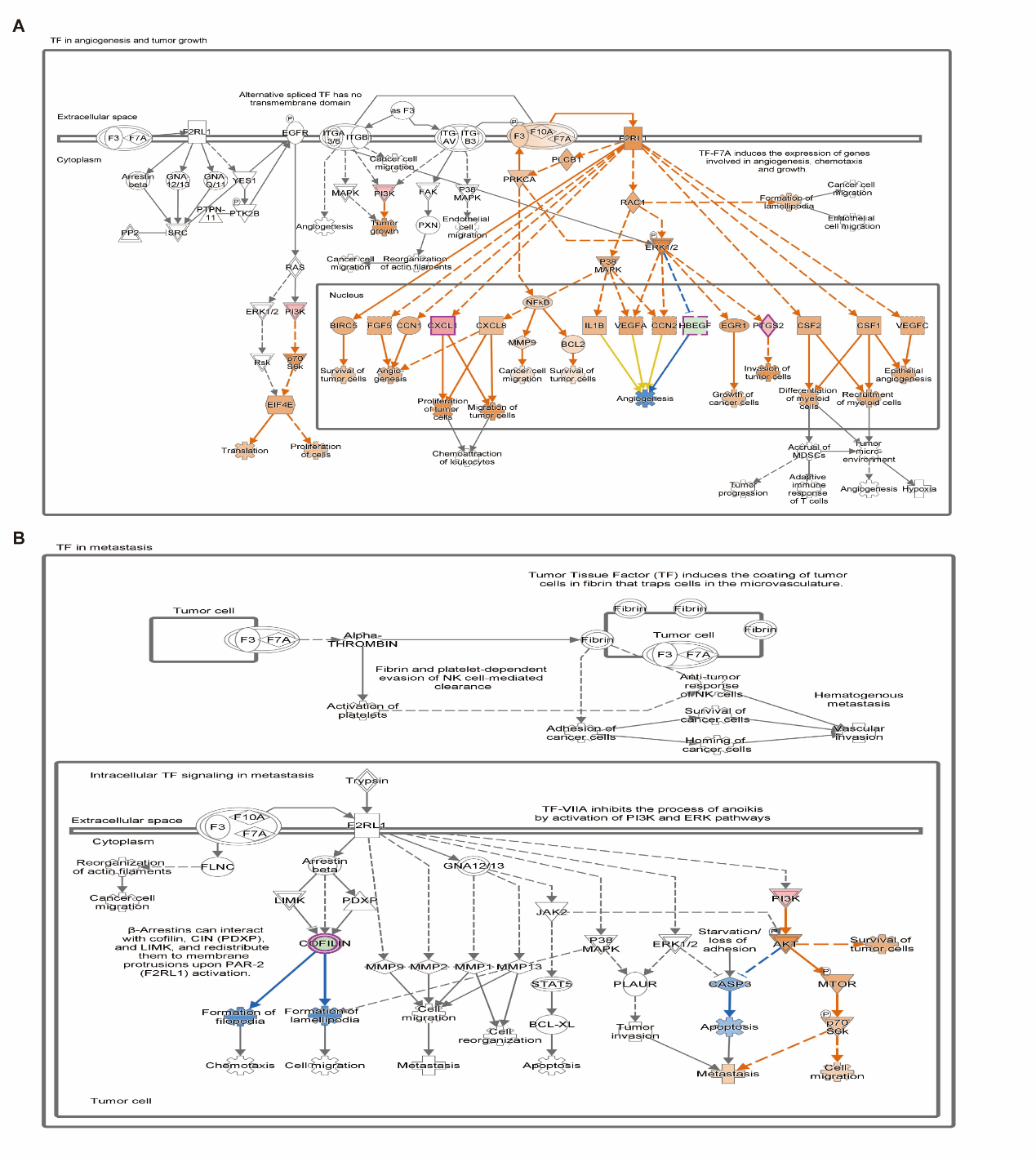


**Fig S4. Roles of tissue factor in cancer.**

A TF in angiogenesis and tumor growth.

B TF in metastasis.

**
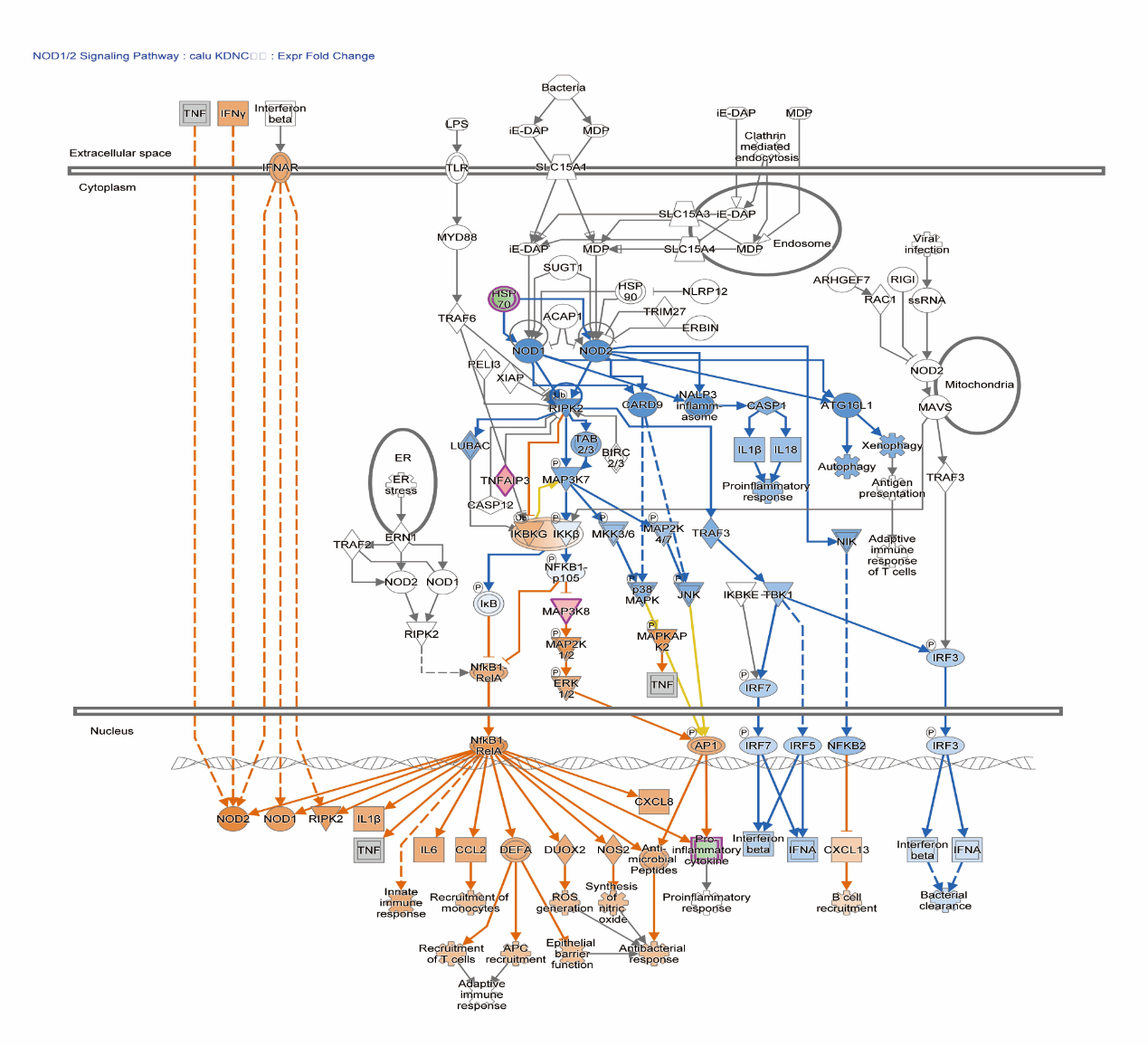
**

**Fig S5. NOD1/2 signaling pathway.**

**
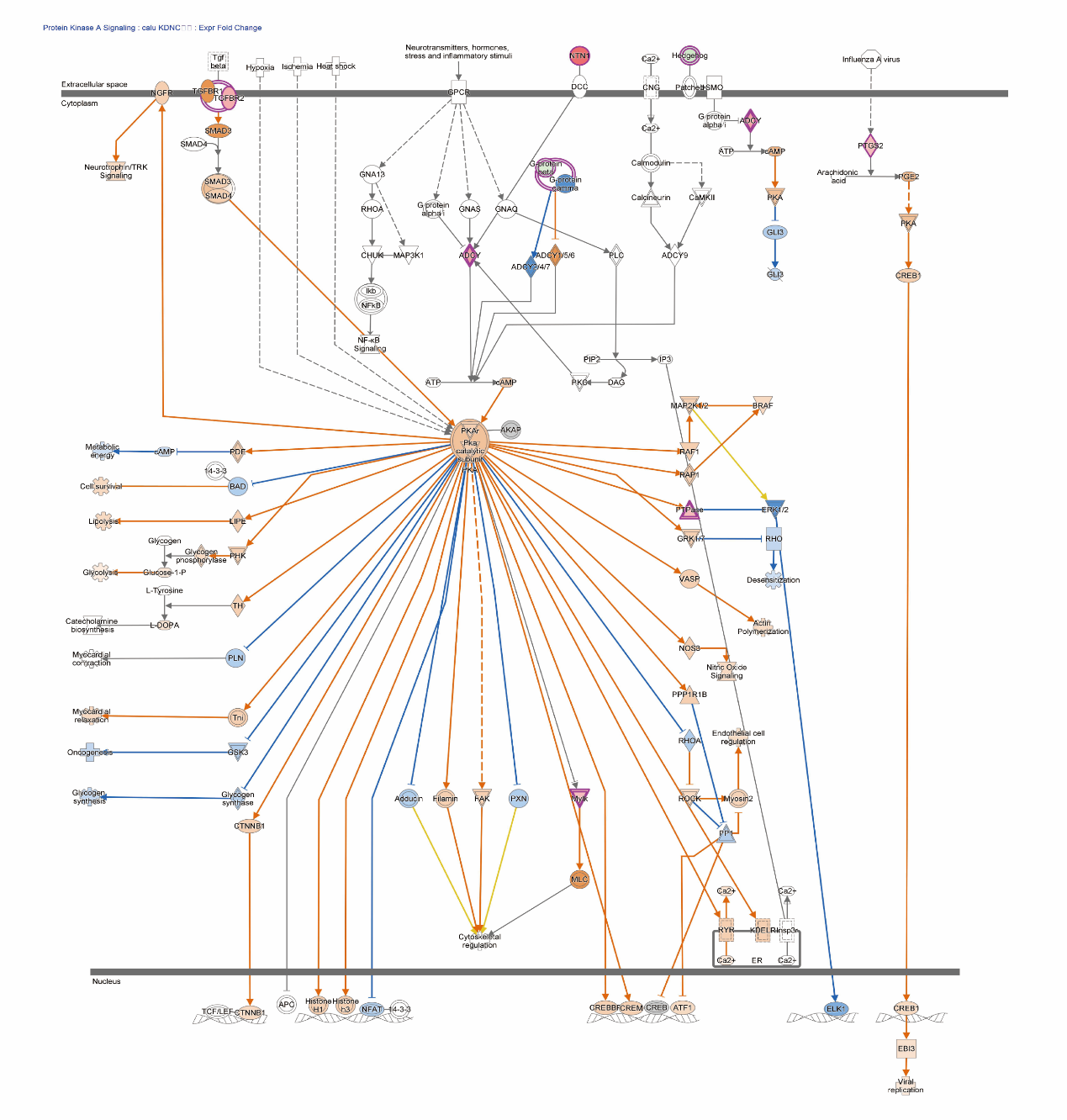
**

**Fig S6. Protein kinase A signaling pathway.**
