# Supplementary material for: CALU promotes lung adenocarcinoma progression by enhancing cell proliferation, migration and invasion
Source: Respir Res. 2024 Jul 5;25:267. doi: 10.1186/s12931-024-02901-3 (PMC11227236; doi:10.1186/s12931-024-02901-3)

Fig2

calumenin- HBE-A549-H1299-PC9-H838-H1975-95D-H292





gapdh- HBE-A549-H1299-PC9-H838-H1975-95D-H292





Fig3

CALU-KD-A549


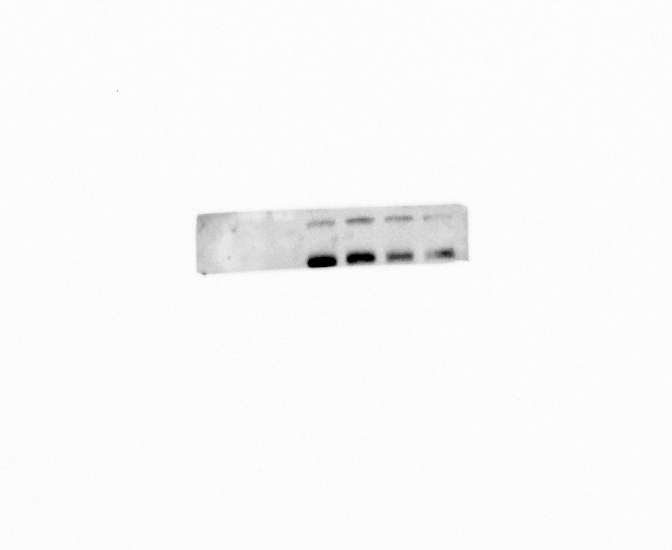


GAPDH-KD-A549


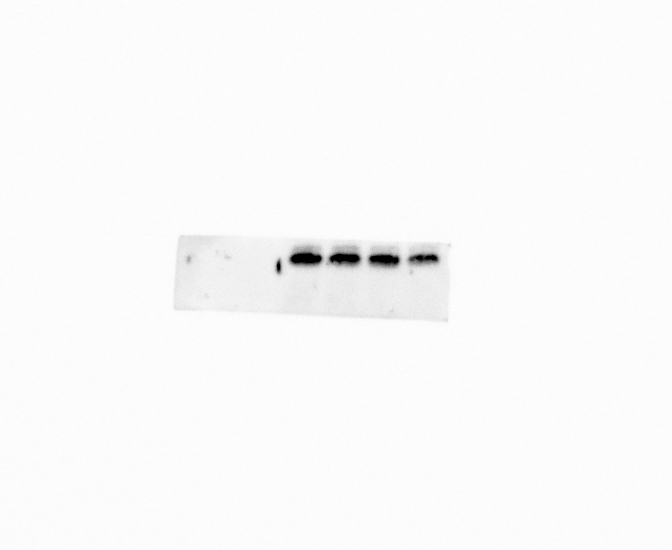


CALU-OE-A549





GAPDH-OE-A549


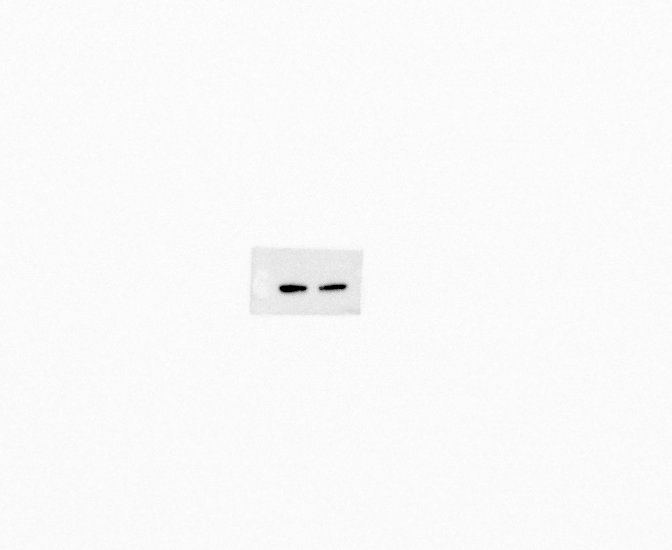


Fig S2

CALU-H1299-NC-KD2


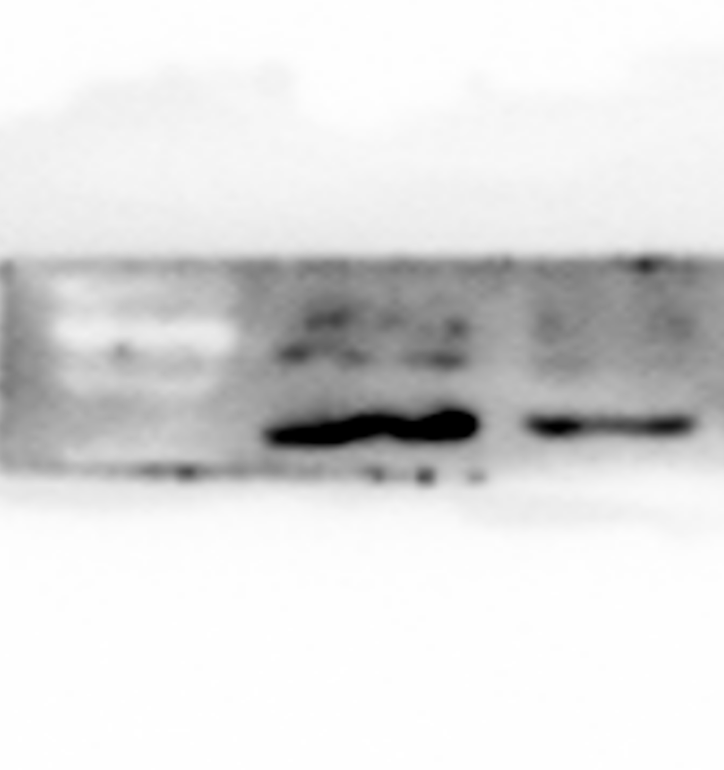


GAPDH-H1299-NC-KD2


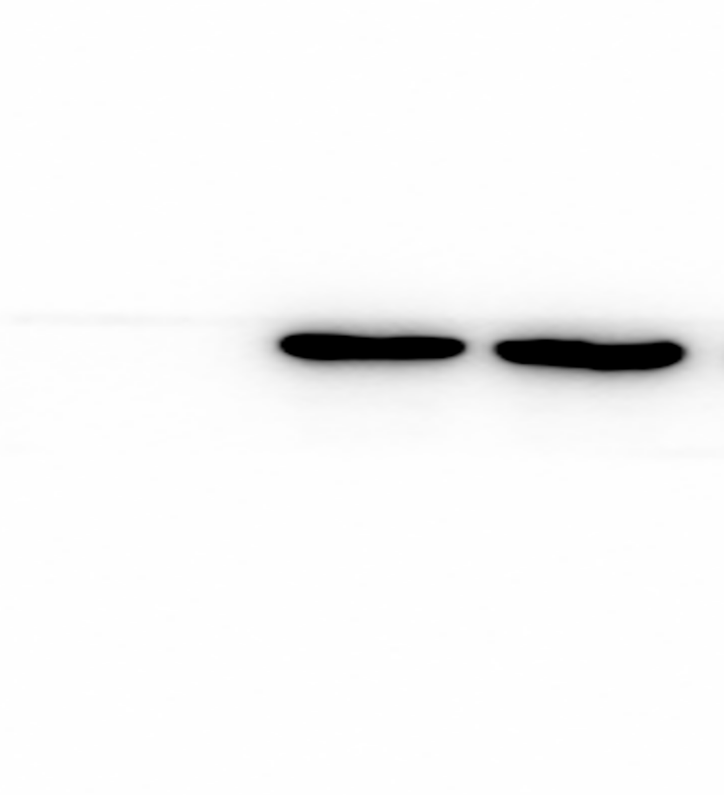


CALU-H1299-NO-OE


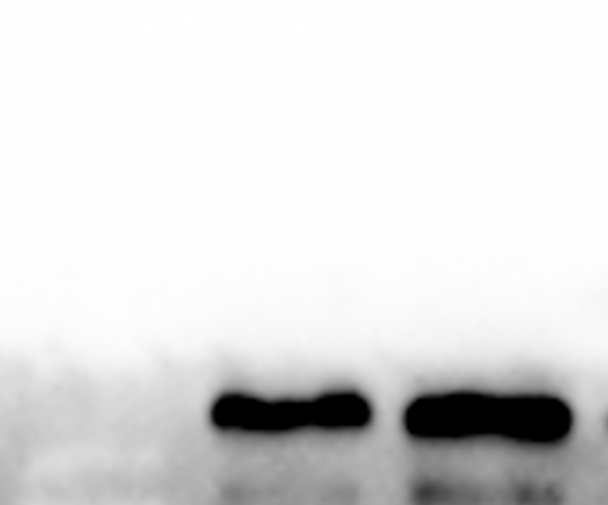


GAPDH-H1299-NO-OE


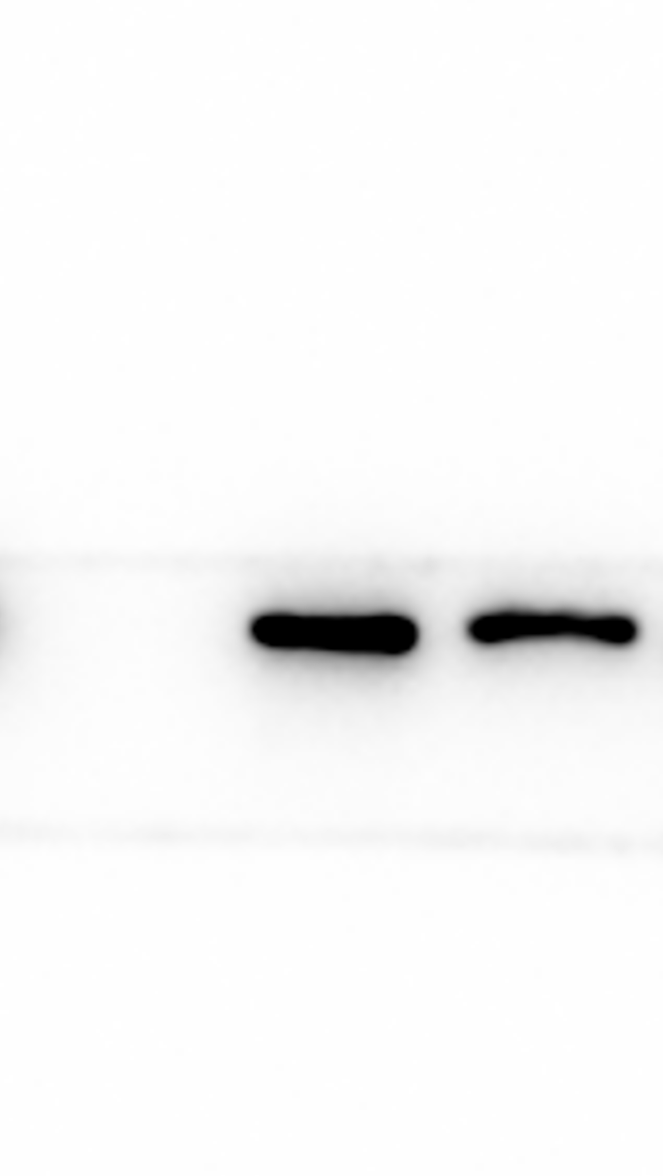

Supplement: Supplementary file 3 — Supplementary Material 3 [file 12931_2024_2901_MOESM3_ESM.docx]
